# Supplementary figures and images for: Cell cycle-dependent and independent mating blocks ensure fungal zygote survival and ploidy maintenance
Source: PLoS Biol. 2021 Jan 6;19(1):e3001067. doi: 10.1371/journal.pbio.3001067 (PMC7815208; doi:10.1371/journal.pbio.3001067)

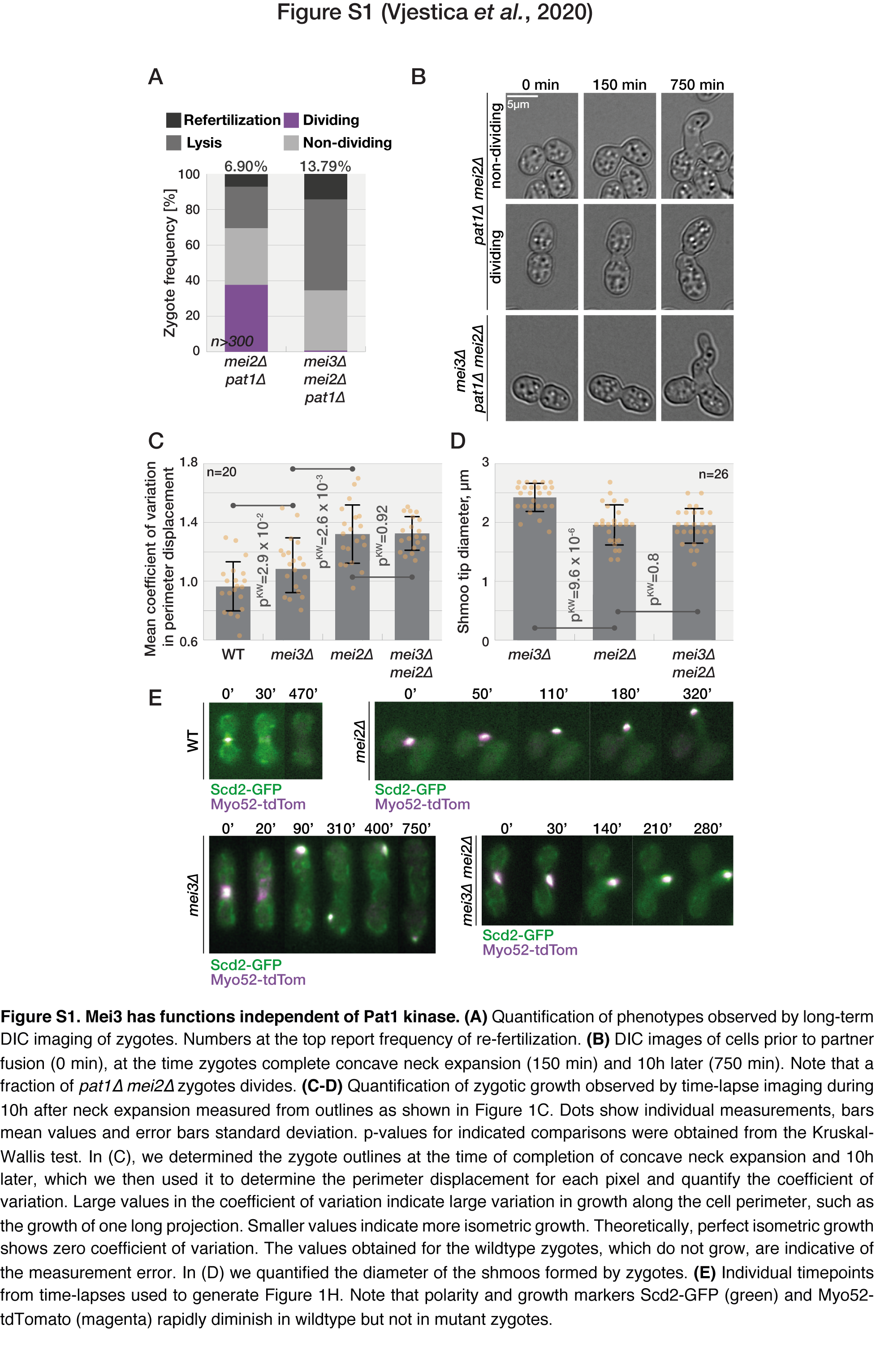

Supplement: S1 Fig — (A) Quantification of phenotypes observed by long-term DIC imaging of zygotes. Numbers at the top report frequency of refertilization. (B) DIC images of cells prior to partner fusion (0 minute), at the time zygotes complete concave neck expansion (150 minutes), and 10 hours later (750 minutes). Note that a fraction of pat1Δ mei2Δ zygotes divides. (C and D) Quantification of zygotic growth observed by time-lapse imaging during 10 hours after neck expansion measured from outlines as shown in Fig 1C. Dots show individual measurements, bars mean values, and error bars standard deviation. p-Values for indicated comparisons were obtained from the Kruskal–Wallis test. In (C), we determined the zygote outlines at the time of completion of concave neck expansion and 10 hours later, which we then used it to determine the perimeter displacement for each pixel and quantify the coefficient of variation. Large values in the coefficient of variation indicate large variation in growth along the cell perimeter, such as the growth of 1 long projection. Smaller values indicate more isometric growth. Theoretically, perfect isometric growth shows 0 coefficient of variation. The values obtained for the wild-type zygotes, which do not grow, are indicative of the measurement error. In (D), we quantified the diameter of the shmoos formed by zygotes. (E) Individual time points from time lapses used to generate Fig 1H. Note that polarity and growth markers Scd2-GFP (green) and Myo52-tdTomato (magenta) rapidly diminish in wild-type but not in mutant zygotes. The data underlying this Figure may be found at https://doi.org/10.6084/m9.figshare.13274837.v1. (TIF) [file pbio.3001067.s001.tif]

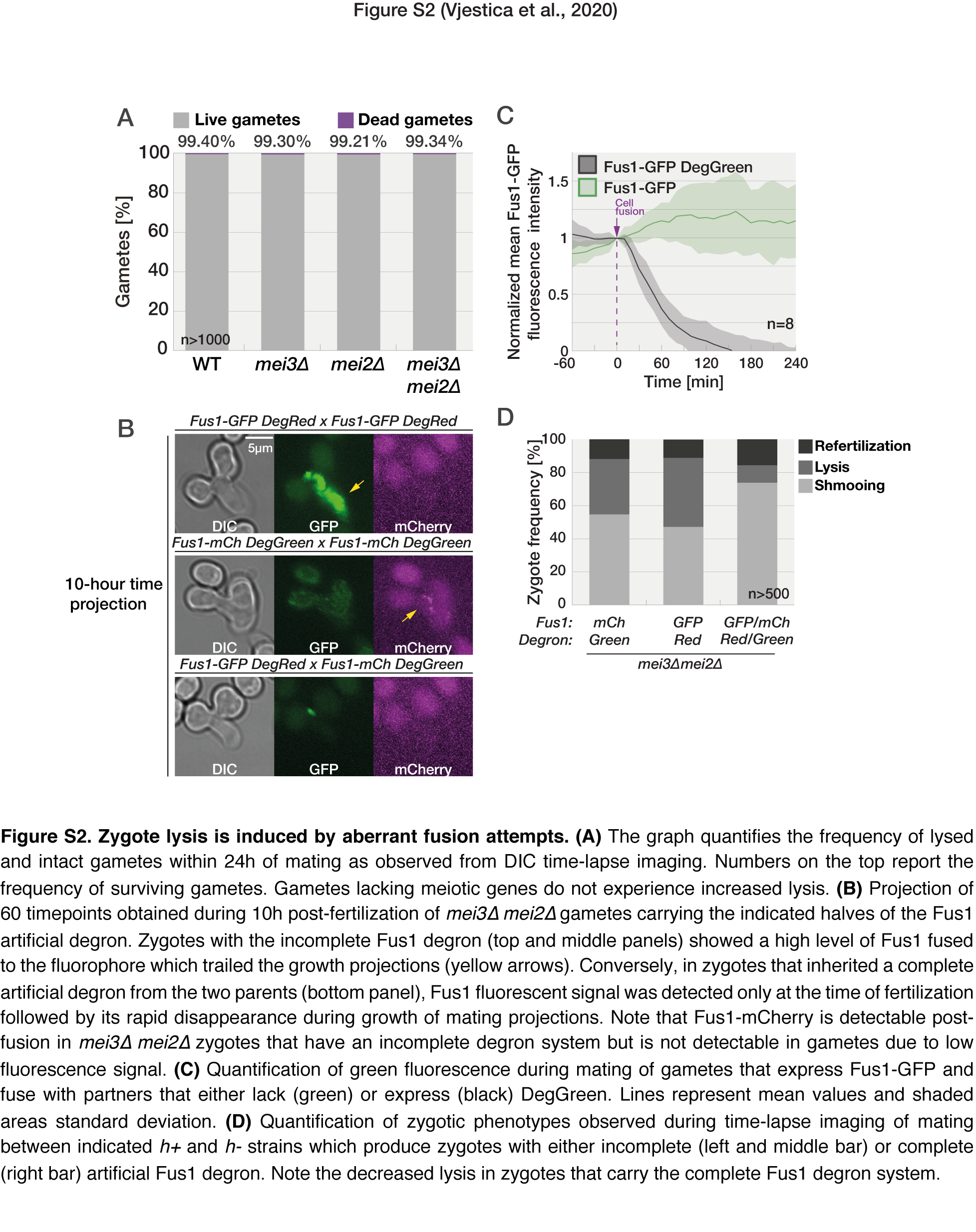

Supplement: S2 Fig — (A) The graph quantifies the frequency of lysed and intact gametes within 24 hours of mating as observed from DIC time-lapse imaging. Numbers on the top report the frequency of surviving gametes. Gametes lacking meiotic genes do not experience increased lysis. (B) Projection of 60 time points obtained during 10 hours postfertilization of mei3Δ mei2Δ gametes carrying the indicated halves of the Fus1 artificial degron. Zygotes with the incomplete Fus1 degron (top and middle panels) showed a high level of Fus1 fused to the fluorophore which trailed the growth projections (yellow arrows). Conversely, in zygotes that inherited a complete artificial degron from the 2 parents (bottom panel), Fus1 fluorescent signal was detected only at the time of fertilization followed by its rapid disappearance during growth of mating projections. Note that Fus1-mCherry is detectable postfusion in mei3Δ mei2Δ zygotes that have an incomplete degron system but is not detectable in gametes due to low fluorescence signal. (C) Quantification of green fluorescence during mating of gametes that express Fus1-GFP and fuse with partners that either lack (green) or express (black) DegGreen. Lines represent mean values and shaded areas standard deviation. (D) Quantification of zygotic phenotypes observed during time-lapse imaging of mating between indicated h+ and h− strains which produce zygotes with either incomplete (left and middle bar) or complete (right bar) artificial Fus1 degron. Note the decreased lysis in zygotes that carry the complete Fus1 degron system. The data underlying this Figure may be found at https://doi.org/10.6084/m9.figshare.13274837.v1. (TIF) [file pbio.3001067.s002.tif]

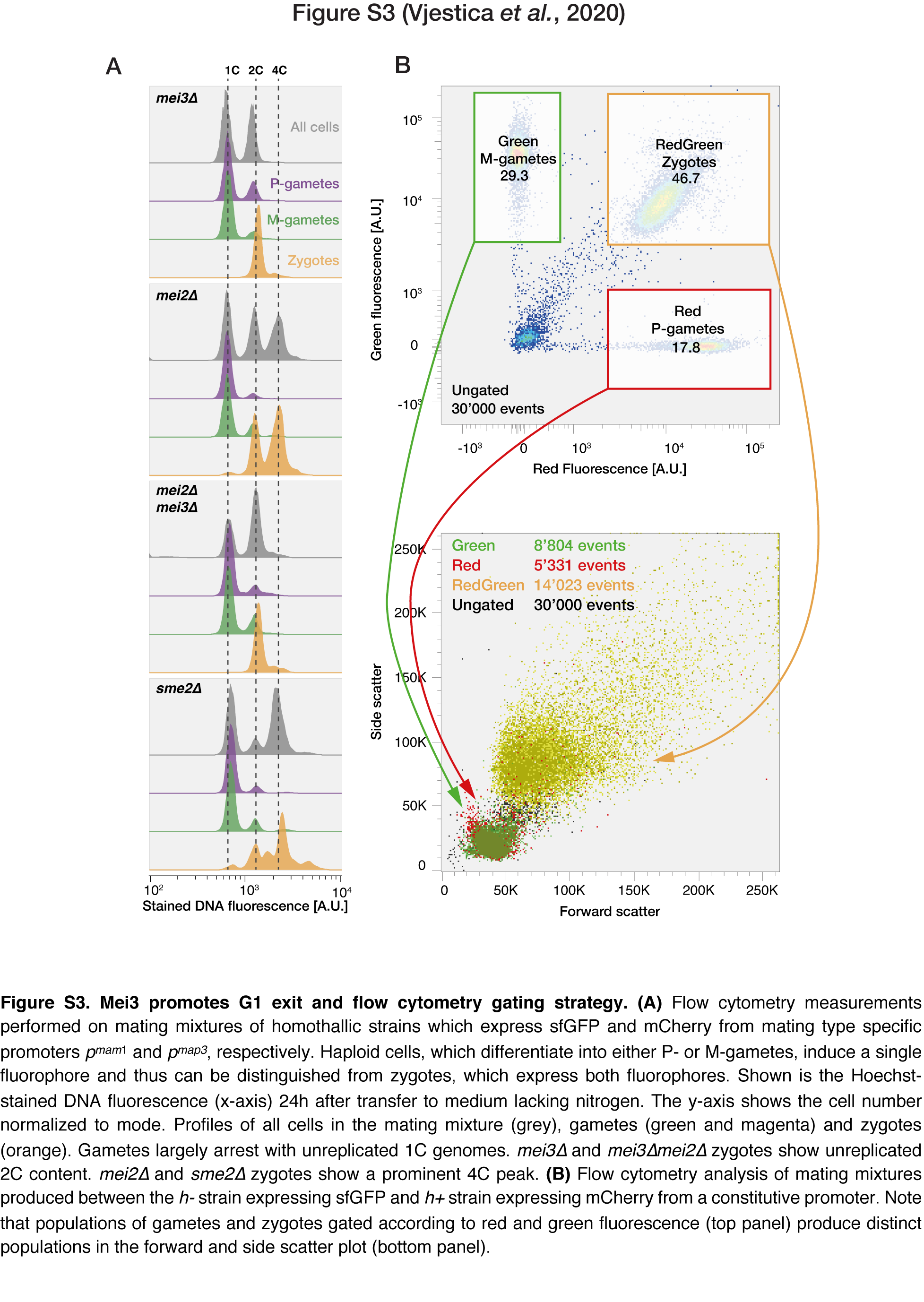

Supplement: S3 Fig — (A) Flow cytometry measurements performed on mating mixtures of homothallic strains which express sfGFP and mCherry from mating type-specific promoters pmam1 and pmap3, respectively. Haploid cells, which differentiate into either P- or M-gametes, induce a single fluorophore and thus can be distinguished from zygotes, which express both fluorophores. Shown is the Hoechst-stained DNA fluorescence (x-axis) 24 hours after transfer to medium lacking nitrogen. The y-axis shows the cell number normalized to mode. Profiles of all cells in the mating mixture (gray), gametes (green and magenta), and zygotes (orange). Gametes largely arrest with unreplicated 1C genomes. mei3Δ and mei3Δmei2Δ zygotes show unreplicated 2C content. mei2Δ and sme2Δ zygotes show a prominent 4C peak. (B) Flow cytometry analysis of mating mixtures produced between the h− strain expressing sfGFP and h+ strain expressing mCherry from a constitutive promoter. Note that populations of gametes and zygotes gated according to red and green fluorescence (top panel) produce distinct populations in the forward and side scatter plot (bottom panel). The data underlying this Figure may be found at https://doi.org/10.6084/m9.figshare.13274837.v1. (TIF) [file pbio.3001067.s003.tif]

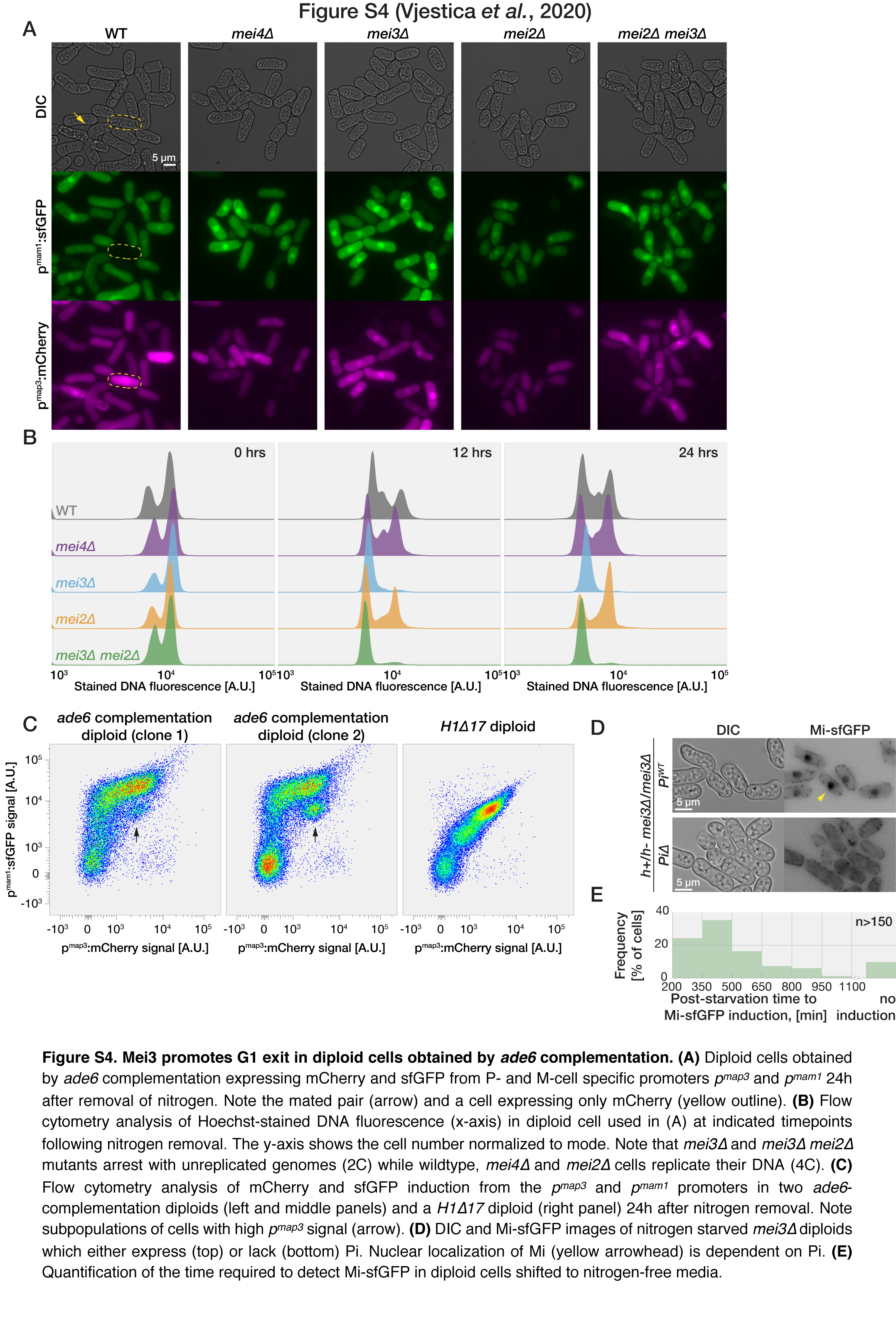

Supplement: S4 Fig — (A) Diploid cells obtained by ade6 complementation expressing mCherry and sfGFP from P- and M-cell-specific promoters pmap3 and pmam1 24 hours after removal of nitrogen. Note the mated pair (arrow) and a cell expressing only mCherry (yellow outline). (B) Flow cytometry analysis of Hoechst-stained DNA fluorescence (x-axis) in diploid cell used in (A) at indicated time points following nitrogen removal. The y-axis shows the cell number normalized to mode. Note that mei3Δ and mei3Δ mei2Δ mutants arrest with unreplicated genomes (2C), while wild-type, mei4Δ, and mei2Δ cells replicate their DNA (4C). (C) Flow cytometry analysis of mCherry and sfGFP induction from the pmap3 and pmam1 promoters in 2 ade6-complementation diploids (left and middle panels) and an H1Δ17 diploid (right panel) 24 hours after nitrogen removal. Note subpopulations of cells with high pmap3 signal (arrow). (D) DIC and Mi-sfGFP images of nitrogen-starved mei3Δ diploids which either express (top) or lack (bottom) Pi. Nuclear localization of Mi (yellow arrowhead) is dependent on Pi. (E) Quantification of the time required to detect Mi-sfGFP in diploid cells shifted to nitrogen-free media. The data underlying this Figure may be found at https://doi.org/10.6084/m9.figshare.13274837.v1. (TIF) [file pbio.3001067.s004.tif]

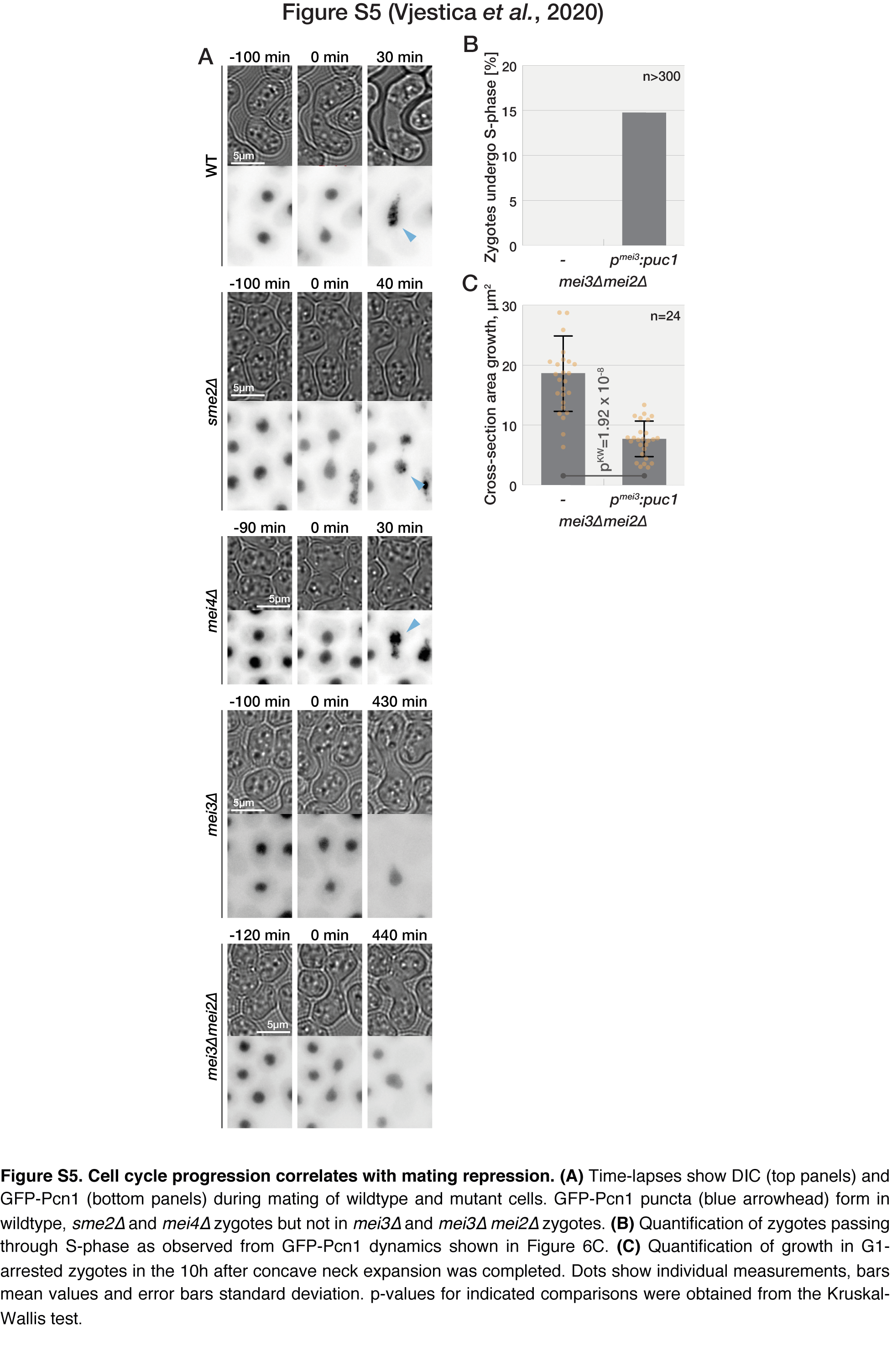

Supplement: S5 Fig — (A) Time lapses show DIC (top panels) and GFP-Pcn1 (bottom panels) during mating of wild-type and mutant cells. GFP-Pcn1 puncta (blue arrowhead) form in wild-type, sme2Δ, and mei4Δ zygotes but not in mei3Δ and mei3Δ mei2Δ zygotes. (B) Quantification of zygotes passing through S-phase as observed from GFP-Pcn1 dynamics shown in Fig 6C. (C) Quantification of growth in G1-arrested zygotes in the 10 hours after concave neck expansion was completed. Dots show individual measurements, bars mean values, and error bars standard deviation. p-Values for indicated comparisons were obtained from the Kruskal–Wallis test. The data underlying this Figure may be found at https://doi.org/10.6084/m9.figshare.13274837.v1. (TIF) [file pbio.3001067.s005.tif]

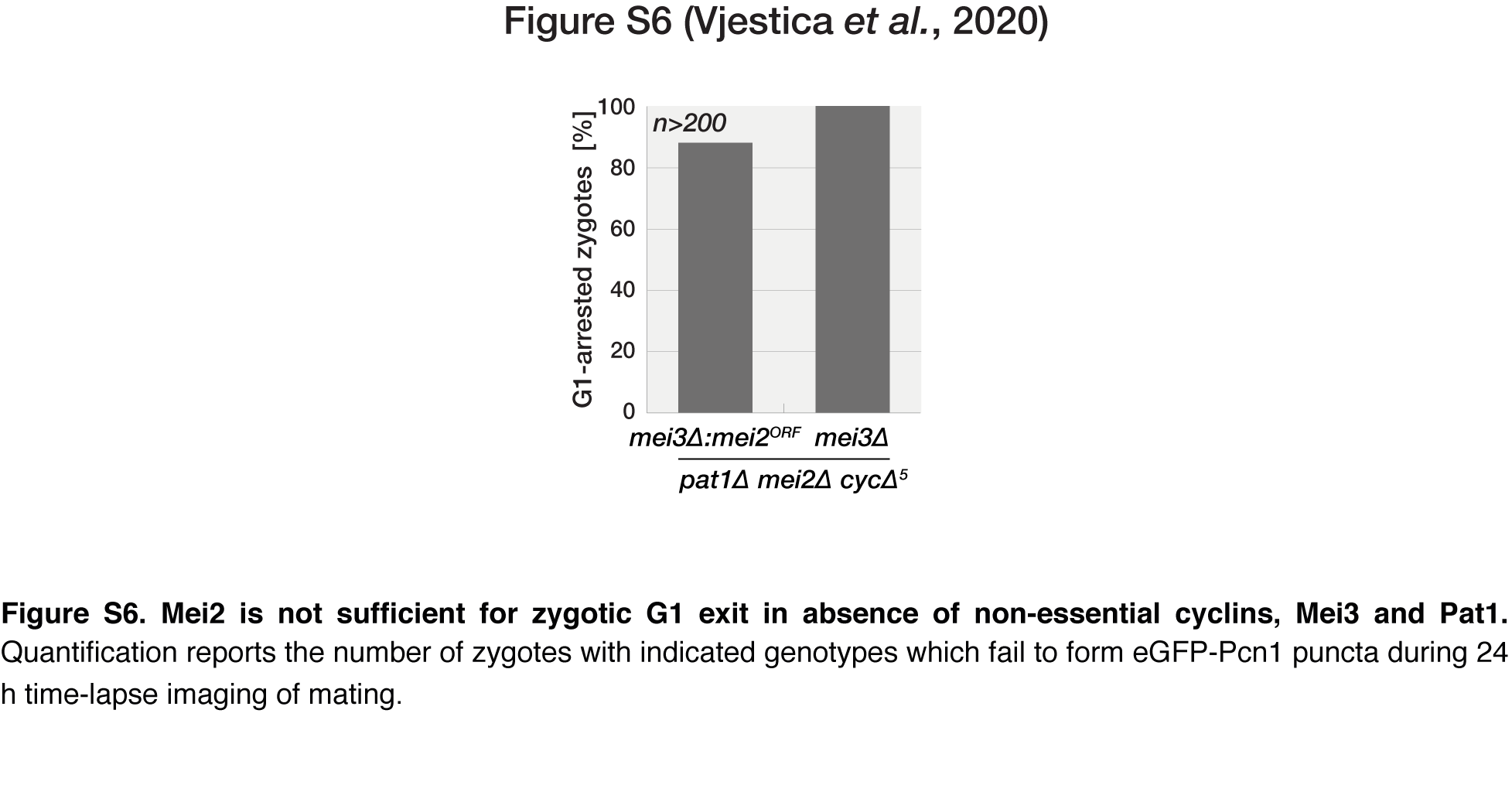

Supplement: S6 Fig — Quantification reports the number of zygotes with indicated genotypes which fail to form eGFP-Pcn1 puncta during 24-hour time-lapse imaging of mating. The data underlying this Figure may be found at https://doi.org/10.6084/m9.figshare.13274837.v1. (TIF) [file pbio.3001067.s006.tif]
