# Supplementary material for: Cell cycle-dependent and independent mating blocks ensure fungal zygote survival and ploidy maintenance
Source: PLoS Biol. 2021 Jan 6;19(1):e3001067. doi: 10.1371/journal.pbio.3001067 (PMC7815208; doi:10.1371/journal.pbio.3001067)
Supplement: S1 Table — (PDF) [file pbio.3001067.s021.pdf]

Table S1

			Note: All prototrophic loci are wildtype unless otherwise indicated.
**Strain ID**	**Genotype**		**Relates to data presented in**
**AV0262**	h+ fus1Δ::natMX	Vjestica *et al.*, 2018	2D 2E
**AV0465**	h90 mei3Δ::kanMX	Vjestica *et al.*, 2018	1C 1F 1G 3B S1C S1D S2A M1
**AV0519**	h90 mei2Δ::kanMX p^{mam2}:GFP::ura4+::mam2+	Vjestica *et al.*, 2018	1B
**AV0521**	h90 mei2Δ::kanMX	Vjestica *et al.*, 2018	1C 1F 1G 3B 3D S1C S1D S2A M1
**AV0526**	h90 mei3Δ::kanMX p^{mam2}:GFP::ura4+::mam2+	Vjestica *et al.*, 2018	1B
**AV1152**	h90 mei2^{F644A}::hphMX	This study	3B
**AV1192**	h- ura4+::p^{tdh1*}:sfGFP:terminator^{tdh1}	This study	S3B
**AV1197**	h+ ura4+::p^{tdh1*}:mCherry:terminator^{tdh1}	This study	S3B
**AV1443**	h+::Mat1[WT]H1^{Δ17}::natMX/h-::Mat1[Mi-sfGFP]H1^{Δ17}::kanMX mei3Δ::mCherry::hphMX/mei3Δ::mCherry::hphMX	This study	3G 3H S4D S4E M7
**AV1444**	h+::Mat1[PiΔ]H1^{Δ17}::natMX/h-::Mat1[Mi-sfGFP]H1^{Δ17}::kanMX mei3Δ::mCherry::hphMX/mei3Δ::mCherry::hphMX	This study	S4D M7
**AV1460**	h90 pat1Δ::hphMX mei2Δ::kanMX	This study	3B 3D S1A S1B M3
**AV1465**	h90 mei2Δ::hphMX pcp1-mCherry::kanMX uch2-mCherry::natMX leu1-32::p^{SV40}:GFP-atb2::leu1+	This study	1E
**AV1504**	h90 mei2Δ::kanMX mei3Δ::kanMX	Vjestica *et al.*, 2018	1C 1F 1G 3B S1C S1D S2A M1
**AV1510**	h90 pat1Δ::natMX mei2Δ::kanMX mei3Δ::kanMX	This study	3B S1A S1B M3
**AV1528**	h90 mei2Δ::kanMX ade6+::p^{tdh1}:mei3:term^{ScADH1}::natMX	This study	3D
**AV1529**	h90 pat1Δ::hphMX mei2Δ::kanMX ade6+::p^{tdh1}:mei3:term^{ScADH1}::natMX	This study	3D
**AV1581**	h90 mei2^{F644A}::hphMX mei3Δ::kanMX	This study	3B
**AV1587**	h- mei2Δ::hphMX	This study	3C
**AV1629**	h- mei2Δ::hphMX ura4+::p^{tdh1*}:mCherry:terminator^{tdh1}	This study	3A
**AV1632**	h- mei3Δ::kanMX ura4+::p^{tdh1*}:sfGFP:terminator^{tdh1}	This study	3A
**AV1645**	h90 mei2Δ::kanMX lys3+::p^{map3}:mCherry::natMX rlc1-sfGFP::natMX	This study	1D M2
**AV1646**	h90 mei3Δ::kanMX lys3+::p^{map3}:mCherry::natMX rlc1-sfGFP::natMX	This study	M2
**AV1647**	h90 mei2Δ::kanMX mei3Δ::kanMX lys3+::p^{map3}:mCherry::natMX rlc1-sfGFP::natMX	This study	M2
**AV1659**	h+ mei2Δ::hphMX ura4+::p^{tdh1*}:sfGFP:terminator^{tdh1}	This study	3A
**AV1660**	h+ mei3Δ::kanMX ura4+::p^{tdh1*}:mCherry:terminator^{tdh1}	This study	3A
**AV1662**	h+ mei2Δ::hphMX mei3Δ::kanMX ura4+::p^{tdh1*}:sfGFP:terminator^{tdh1}	This study	3A
**AV1664**	h- mei2Δ::hphMX mei3Δ::kanMX ura4+::p^{tdh1*}:mCherry:terminator^{tdh1}	This study	3A
**AV1686**	h- mei4Δ::ura4+ ura4+::p^{tdh1*}:mCherry:terminator^{tdh1}	This study	3A
**AV1689**	h+ mei4Δ::ura4+ ura4+::p^{tdh1*}:sfGFP:terminator^{tdh1}	This study	3A
**AV1719**	h90 mei3Δ::kanMX ade6+::p^{mam1*}:sfGFP:terminator^{ScADH1}::bsdMX::p^{map3}:mCherry:terminator^{ScADH1}	This study	S3A
**AV1720**	h90 mei2Δ::kanMX ade6+::p^{mam1*}:sfGFP:terminator^{ScADH1}::bsdMX::p^{map3}:mCherry:terminator^{ScADH1}	This study	S3A
**AV1722**	h90 sme2Δ::ura4+ ade6+::p^{mam1*}:sfGFP:terminator^{ScADH1}::bsdMX::p^{map3}:mCherry:terminator^{ScADH1}	This study	S3A
**AV1723**	h90 mei2Δ::kanMX mei3Δ::kanMX ade6+::p^{mam1*}:sfGFP:terminator^{ScADH1}::bsdMX::p^{map3}:mCherry:terminator^{ScADH1}	Vjestica *et al.*, 2018	1B S3A
**AV1725**	h90 ura4+::p^{pcn1}:eGFP-linker-pcn1:3'UTR^{pcn1}:terminator^{NMT}::natMX	This study	3E 3F S5A M9
**AV1726**	h90 mei2Δ::kanMX ura4+::p^{pcn1}:eGFP-linker-pcn1:3'UTR^{pcn1}:terminator^{NMT}::natMX	This study	3E 3F 6A 6B M9 M10
**AV1727**	h90 mei3Δ::kanMX ura4+::p^{pcn1}:eGFP-linker-pcn1:3'UTR^{pcn1}:terminator^{NMT}::natMX	This study	S5A M9
**AV1728**	h90 mei2Δ::kanMX mei3Δ::kanMX ura4+::p^{pcn1}:eGFP-linker-pcn1:3'UTR^{pcn1}:terminator^{NMT}::natMX	This study	6C 6D S5A S5B S5C M9 M11
**AV1729**	h90 mei4Δ::ura4+ ura4+::p^{pcn1}:eGFP-linker-pcn1:3'UTR^{pcn1}:terminator^{nmt}::natMX	This study	S5A M9

**AV1730**	h90 sme2Δ::ura4+ ura4+::p^{pcn1}:eGFP-linker-pcn1:3'UTR^{pcn1}:$terminator^{nmt}$::natMX	This study	S5A M9
**AV1755**	h+/h- lys3+::p^{map3}:mCherry::natMX/lys3+::p^{map3}:mCherry::natMX ade6-M216::p^{mam1*}:sfGFP:$terminator^{ScADH1}$::bsdMX/ade6-M210::p^{mam1*}:sfGFP:$terminator^{ScADH1}$::bsdMX	This study	S4A S4B S4C
**AV1756**	h+/h- lys3+::p^{map3}:mCherry::natMX/lys3+::p^{map3}:mCherry::natMX ade6-M216::p^{mam1*}:sfGFP:$terminator^{ScADH1}$::bsdMX/ade6-M210::p^{mam1*}:sfGFP:$terminator^{ScADH1}$::bsdMX	This study	S4C
**AV1757**	h+/h- mei2Δ::hphMX/mei2Δ::hphMX lys3+::p^{map3}:mCherry::natMX/lys3+::p^{map3}:mCherry::natMX ade6-M216::p^{mam1*}:sfGFP:$terminator^{ScADH1}$::bsdMX/ade6-M210::p^{mam1*}:sfGFP:$terminator^{ScADH1}$::bsdMX	This study	S4A S4B
**AV1758**	h+/h- mei3Δ::kanMX/mei3Δ::kanMX lys3+::p^{map3}:mCherry::natMX/lys3+::p^{map3}:mCherry::natMX ade6-M216::p^{mam1*}:sfGFP:$terminator^{ScADH1}$::bsdMX/ade6-M210::p^{mam1*}:sfGFP:$terminator^{ScADH1}$::bsdMX	This study	S4A S4B
**AV1759**	h+/h- mei2Δ::hphMX/mei2Δ::hphMX mei3Δ::kanMX/mei3Δ::kanMX lys3+::p^{map3}:mCherry::natMX/lys3+::p^{map3}:mCherry::natMX ade6-M216::p^{mam1*}:sfGFP:$terminator^{ScADH1}$::bsdMX/ade6-M210::p^{mam1*}:sfGFP:$terminator^{ScADH1}$::bsdMX	This study	S4A S4B
**AV1760**	h+/h- mei4Δ::ura4+/mei4Δ::ura4+ lys3+::p^{map3}:mCherry::natMX/lys3+::p^{map3}:mCherry::natMX ade6-M216::p^{mam1*}:sfGFP:$terminator^{ScADH1}$::bsdMX/ade6-M210::p^{mam1*}:sfGFP:$terminator^{ScADH1}$::bsdMX	This study	S4A S4B
**AV1761**	h+::Mat1[WT]$H1^{\Delta17}$::bleMX/h-::Mat1[WT]$H1^{\Delta17}$::natMX ade6+::p^{mam1*}:sfGFP:$terminator^{ScADH1}$::bsdMX::p^{map3}:mCherry:$terminator^{ScADH1}$/ade6+::p^{mam1*}:sfGFP:$terminator^{ScADH1}$::bsdMX::p^{map3}:mCherry:$terminator^{ScADH1}$	This study	3A 3B 3F M6
**AV1762**	h+::Mat1[WT]$H1^{\Delta17}$::bleMX/h-::Mat1[WT]$H1^{\Delta17}$::natMX mei2Δ::kanMX/mei2Δ::kanMX ade6+::p^{mam1*}:sfGFP:$terminator^{ScADH1}$::bsdMX::p^{map3}:mCherry:$terminator^{ScADH1}$/ade6+::p^{mam1*}:sfGFP:$terminator^{ScADH1}$::bsdMX::p^{map3}:mCherry:$terminator^{ScADH1}$	This study	3A 3B 3C 3F M6
**AV1763**	h+::Mat1[WT]$H1^{\Delta17}$::bleMX/h-::Mat1[WT]$H1^{\Delta17}$::natMX mei3Δ::kanMX/mei3Δ::kanMX ade6+::p^{mam1*}:sfGFP:$terminator^{ScADH1}$::bsdMX::p^{map3}:mCherry:$terminator^{ScADH1}$/ade6+::p^{mam1*}:sfGFP:$terminator^{ScADH1}$::bsdMX::p^{map3}:mCherry:$terminator^{ScADH1}$	This study	3A 3B 3D 3F M6
**AV1764**	h+::Mat1[WT]$H1^{\Delta17}$::bleMX/h-::Mat1[WT]$H1^{\Delta17}$::natMX mei2Δ::kanMX/mei2Δ::kanMX mei3Δ::kanMX/mei3Δ::kanMX ade6+::p^{mam1*}:sfGFP:$terminator^{ScADH1}$::bsdMX::p^{map3}:mCherry:$terminator^{ScADH1}$/ade6+::p^{mam1*}:sfGFP:$terminator^{ScADH1}$::bsdMX::p^{map3}:mCherry:$terminator^{ScADH1}$	This study	3A 3B 3E 3F M6
**AV1765**	h+::Mat1[WT]$H1^{\Delta17}$::bleMX/h-::Mat1[WT]$H1^{\Delta17}$::natMX mei4Δ::ura4+/mei4Δ::ura4+ ade6+::p^{mam1*}:sfGFP:$terminator^{ScADH1}$::bsdMX::p^{map3}:mCherry:$terminator^{ScADH1}$/ade6+::p^{mam1*}:sfGFP:$terminator^{ScADH1}$::bsdMX::p^{map3}:mCherry:$terminator^{ScADH1}$	This study	3A 3B
**AV1785**	h90 scd2-GFP::hphMX myo52-tdTomato::natMX	This study	1H S1E M4
**AV1803**	h90 mei2Δ::kanMX scd2-GFP::hphMX myo52-tdTomato::natMX	This study	1H S1E 1I M4
**AV1804**	h90 mei2Δ::kanMX mei3Δ::kanMX scd2-GFP::hphMX myo52-tdTomato::natMX	This study	1H S1E 1I M4
**AV1814**	h90 mei3Δ::kanMX scd2-GFP::hphMX myo52-tdTomato::natMX	This study	1H S1E 1I M4
**AV1829**	h90 mei3Δ::$mei2^{ORF}$::hphMX mei2Δ::hphMX pat1Δ::hphMX	This study	5C
**AV1830**	h+ mei3Δ::$mei2^{ORF}$::hphMX mei2Δ::hphMX pat1Δ::hphMX	This study	5B 5I
**AV1831**	h- mei3Δ::$mei2^{ORF}$::hphMX mei2Δ::hphMX pat1Δ::hphMX	This study	5B 5I
**AV1982**	h- cig1Δ::bsdMX cig2Δ::natMX puc1Δ::bleMX rem1Δ::kanMX crs1Δ::kanMX mei3Δ::$mei2^{ORF}$::hphMX mei2Δ::hphMX pat1Δ::hphMX	This study	5I 5J 7A 7B 7C M13
**AV1997**	h+ cig1Δ::bsdMX cig2Δ::natMX puc1Δ::bleMX crs1Δ::kanMX rem1Δ::kanMX mei3Δ::$mei2^{ORF}$::hphMX mei2Δ::hphMX pat1Δ::hphMX	This study	5I 5J 7A 7B 7C M13
**AV2016**	h+ cig1Δ::bsdMX cig2Δ::natMX puc1Δ::bleMX crs1Δ::kanMX rem1Δ::kanMX mei3Δ::hphMX mei2Δ::hphMX pat1Δ::hphMX	This study	7A 7B 7C M13

**AV2038**	h- cig1Δ::bsdMX cig2Δ::natMX puc1Δ::bleMX crs1Δ::kanMX rem1Δ::kanMX	This study	5I 5J 6E 6F M12
**AV2050**	h+ cig1Δ::bsdMX cig2Δ::natMX puc1Δ::bleMX crs1Δ::kanMX rem1Δ::kanMX	This study	5I 5J 6E 6F M12
**AV2104**	h- aha1+::kanMX::p^{tdh1}:mCherry::SPBC1711.09c+	This study	5D
**AV2105**	h+ leu1+::kanMX::p^{tdh1}:sfGFP::apc10+	This study	5D
**AV2106**	h- aha1+::kanMX::p^{tdh1}:mCherry::SPBC1711.09c+ mei3Δ::mei2^{ORF}::hphMX mei2Δ::hphMX pat1Δ::hphMX	This study	5D
**AV2107**	h+ leu1+::kanMX::p^{tdh1}:sfGFP::apc10+ mei3Δ::mei2^{ORF}::hphMX mei2Δ::hphMX pat1Δ::hphMX	This study	5D
**AV2178**	h- ura4+::p^{tdh1}:Pof1^{Nterminus}-GBP:terminator^{ScCYC1}	This study	2D
**AV2186**	h- ura4+::p^{tdh1}:Pof1^{Nterminus}-ChBP:terminator^{ScCYC1}	This study	2E
**AV2210**	h- ura4+::p^{pcn1}:eGFP-linker-pcn1:3'UTR^{pcn1}:terminator^{nmt}::bsdMX aha1+::kanMX::p^{tdh1}:mCherry::SPBC1711.09c+ mei3Δ::mei2^{ORF}::hphMX mei2Δ::hphMX pat1Δ::hphMX	This study	5E 5F M8
**AV2212**	h+ ura4+::p^{pcn1}:eGFP-linker-pcn1:3'UTR^{pcn1}:terminator^{nmt}::bsdMX mei3Δ::mei2^{ORF}::hphMX mei2Δ::hphMX pat1Δ::hphMX	This study	5E 5F M8
**AV2216**	h+ ura4+::p^{pcn1}:eGFP-linker-pcn1:3'UTR^{pcn1}:terminator^{nmt}::bsdMX	This study	5E 5F M8
**AV2244**	h- aha1+::kanMX::p^{tdh1}:mCherry::SPBC1711.09c+ ura4+::p^{pcn1}:eGFP-linker-pcn1:3'UTR^{pcn1}:terminator^{nmt}::bsdMX	This study	5E 5F M8
**AV2268**	h- mei2Δ::hphMX ura4+::p^{tdh1*}:mei3:terminator^{tdh1}	This study	3C
**AV2343**	h- wildtype	S.pombe 972 derivative strain	2B 2C 5B 5I 6F
**AV2344**	h+ wildtype	This study	5B 5I 6F
**AV2462**	h- cig1Δ::bsdMX cig2Δ::natMX puc1Δ::bleMX crs1Δ::kanMX rem1Δ::kanMX mei3Δ::mei2^{ORF}::hphMX pat1Δ::hphMX mei2Δ::hphMX ura4+::p^{pcn1}:eGFP-linker-pcn1:3'UTR^{pcn1}:terminator^{nmt}::natMX ade6+ leu1+	This study	S6 M13
**AV2466**	h+ cig1Δ::bsdMX cig2Δ::natMX puc1Δ::bleMX crs1Δ::kanMX rem1Δ::kanMX mei3Δ::mei2^{ORF}::hphMX pat1Δ::hphMX mei2Δ::hphMX ura4+::p^{pcn1}:eGFP-linker-pcn1:3'UTR^{pcn1}:terminator^{nmt}::natMX ade6+ leu1+	This study	S6 M13
**AV2564**	h? ade6+::p^{act1}:mCherry	This study	2C
**AV2565**	h? ade6+::p^{act1}:mCherry ura4+::p^{tdh1}:Pof1Nterminus-ChBP:terminator^{ScCYC1}	This study	2C
**AV2573**	h- cig1Δ::bsdMX cig2Δ::natMX puc1Δ::bleMX crs1Δ::kanMX rem1Δ::kanMX mei3Δ::hphMX mei2Δ::hphMX pat1Δ::hphMX	This study	7A 7B 7C M13
**AV2584**	h90 lys3+::p^{map3}:mCherry::natMX ura4+::p^{mei3}:mei2^{ORF}::hphMX pat1Δ::hphMX mei2Δ::kanMX his5+::p^{pcn1}:eGFP-linker-pcn1:terminator^{NMT}::bleMX	This study	5G 5H
**AV2585**	h90 lys3+::p^{map3}:mCherry::natMX ura4+::p^{mei3}:mei2^{ORF}::hphMX mei3Δ::kanMX pat1Δ::hphMX mei2Δ::kanMX his5+::p^{pcn1}:eGFP-linker-pcn1:terminator^{NMT}::bleMX	This study	5G 5H
**AV2589**	h90 mei2Δ::kanMX mei3Δ:puc1::hphMX ura4+::p^{pcn1}:eGFP-linker-pcn1:3'UTR^{pcn1}:terminator^{NMT}::natMX	This study	6C 6D S5B S5C M11
**AV2605**	h- scd2-eGFP::patMX cig1Δ::bsdMX cig2Δ::natMX puc1Δ::bleMX rem1Δ::kanMX crs1Δ::kanMX mei3Δ::mei2^{ORF}::hphMX mei2Δ::hphMX pat1Δ::hphMX	This study	7D M14
**AV2606**	h+ scd2-eGFP::patMX cig1Δ::bsdMX cig2Δ::natMX puc1Δ::bleMX crs1Δ::kanMX rem1Δ::kanMX mei3Δ::mei2^{ORF}::hphMX mei2Δ::hphMX pat1Δ::hphMX	This study	7D M14
**AV2607**	h+ scd2-eGFP::patMX cig1Δ::bsdMX cig2Δ::natMX puc1Δ::bleMX crs1Δ::kanMX rem1Δ::kanMX mei3Δ::hphMX mei2Δ::hphMX pat1Δ::hphMX	This study	7D M14
**AV2608**	h- scd2-eGFP::patMX cig1Δ::bsdMX cig2Δ::natMX puc1Δ::bleMX crs1Δ::kanMX rem1Δ::kanMX mei3Δ::hphMX mei2Δ::hphMX pat1Δ::hphMX	This study	7D M14
**AV2620**	h? ade6+::p^{act1}:GFP:term^{NMT}	This study	2B
**AV2621**	h? ade6+::p^{act1}:GFP:term^{NMT} ura4+::p^{tdh1}:Pof1Nterminus-GBP:terminator^{ScCYC1}	This study	2B
**AV2624**	h- fus1-GFP::kanMX	This study	2D
**AV2625**	h+ fus1-meGFP::kanMX::p^{tdh1}:Pof1Nterminus-ChBP:term^{ScCYC1} mei3Δ::bsdMX mei2Δ::hphMX	This study	2G S2B S2C S2D M5
**AV2626**	h- fus1-mCherry::kanMX	This study	2E
**AV2629**	h- fus1-GFP::kanMX ura4+::p^{tdh1}:Pof1Nterminus-GBP:terminator^{ScCYC1}	This study	2D

**AV2632**	h- fus1-meGFP::kanMX::p^{tdh1}:Pof1Nterminus-ChBP:$term^{ScCYC1}$ mei3Δ::bsdMX mei2Δ::hphMX	This study	2G S2B S2C S2D M5
**AV2634**	h- fus1-mCherry::kanMX::p^{tdh1}:Pof1Nterminus-GBP:$term^{ScCYC1}$ mei3Δ::bsdMX mei2Δ::hphMX	This study	2G S2B S2C S2D M5
**AV2635**	h+ fus1-mCherry::kanMX::p^{tdh1}:Pof1Nterminus-GBP:$term^{ScCYC1}$ mei3Δ::bsdMX mei2Δ::hphMX	This study	2G S2B S2C S2D M5
**AV2636**	h- cig1Δ::bsdMX cig2Δ::natMX puc1Δ::bleMX crs1Δ::kanMX rem1Δ::kanMX mei3Δ::hphMX mei2Δ::hphMX pat1Δ::hphMX ura4+::p^{pcn1}:eGFP-linker-pcn1:$terminator^{nmt1}$::patMX	This study	S6 M13
**AV2637**	h+ cig1Δ::bsdMX cig2Δ::natMX puc1Δ::bleMX crs1Δ::kanMX rem1Δ::kanMX mei3Δ::hphMX mei2Δ::hphMX pat1Δ::hphMX ura4+::p^{pcn1}:eGFP-linker-pcn1:$terminator^{nmt1}$::patMX	This study	S6 M13
**AV2645**	h- fus1-mCherry::kanMX ura4+::p^{tdh1}:$Pof1^{Nterminus}$-ChBP:$terminator^{ScCYC1}$	This study	2E
**AV2678**	h90 ade6+::p^{tdh1}:mei3-$term^{ScAdh1}$::hphMX mei2Δ::kanMX ura4+::p^{pcn1}:eGFP-linker-pcn1:3'UTR^{pcn1}:$terminator^{NMT}$::natMX	This study	3E 3F
**ySM1396**	h90 wildtype	S.pombe 968 strain	1B 1C 1F 1G S1C S2A
