# Supplementary material for: Cell cycle-dependent and independent mating blocks ensure fungal zygote survival and ploidy maintenance
Source: PLoS Biol. 2021 Jan 6;19(1):e3001067. doi: 10.1371/journal.pbio.3001067 (PMC7815208; doi:10.1371/journal.pbio.3001067)
Supplement: S2 Table — (PDF) [file pbio.3001067.s022.pdf]

Table S2

Genetic Marker	Sequence/Source	Description
ade6-M210::p^{mam1*}*:sfGFP:terminator*^{ScADH1}*::bsdMX*	XhoI/PmeI fragment of **pAV0611** was transformed into strain carrying the mutant *ade6-M210* locus and transformants selected for blasticidin-S resistance and adenine auxotrophy.	sfGFP is expressed from the M-gamete specific *p*^{mam1*} promoter (1751bp upstream START codon, single nucleotide mutated) integrated at the mutant *ade6-M210* locus and linked with the *bsdMX* selection cassette.
ade6-M216::p^{mam1*}*:sfGFP:terminator*^{ScADH1}*::bsdMX*	XhoI/PmeI fragment of **pAV0611** was transformed into strain carrying the mutant *ade6-M216* locus and transformants selected for blasticidin-S resistance and adenine auxotrophy.	sfGFP is expressed from the M-gamete specific *p*^{mam1*} promoter (1751bp upstream START codon, single nucleotide mutated) integrated at the mutant *ade6-M216* locus and linked with *bsdMX* selection cassette.
ade6+::p^{act1}*:GFP:term*^{NMT}	PmeI linearized **pAV0884** was transformed into ade6-D19 strains and selected for prototrophic transformants.	GFP is expressed from the strong *p*^{act1} promoter (822bp upstream START codon) integrated at the *ade6* locus.
ade6+::p^{act1}*:mCherry*	PmeI linearized **pAV0377** was transformed into ade6-D19 strains and selected for prototrophic transformants.	mCherry is expressed from the strong *p*^{act1} promoter (822bp upstream START codon) integrated at the *ade6* locus.
ade6+::p^{mam1*}*:sfGFP:terminator*^{ScADH1}*::bsdMX::p*^{map3}*:mCherry:terminator*^{ScADH1}	PmeI linearized **pAV0614** was used to obtain blasticidin-S resistant transformants.	The *ade6* locus is linked to the construct for cytosolic sfGFP expression from the M-gamete specific *p*^{mam1*} promoter (1751bp upstream START codon, single nucleotide mutated), the *bsdMX* selection cassette and the construct for mCherry expression from the P-gamete specific *p*^{map3} promoter (2063bp upstream START codon).
ade6+::p^{tdh1}*:mei3:term*^{ScADH1}*::natMX*	RsrII/BamHI fragment of **pAV0573** was used to obtain nourseothricin resistant transformants.	Mei3 expression is regulated by the strong, constitutive p^{tdh1} promoter (1000bp upstream START codon) and the budding yeast transcriptional terminator linked to the *ade6* locus.
aha1+::kanMX::p^{tdh1}*:mCherry::SPBC1711.09c+*	AfeI linearized **pAV0707** was used to obtain G418 resistant transformants	mCherry is expressed from the strong *p*^{tdh1} promoter, the construct linked with *kanMX* selection cassette and integrated into the intergenic region between *aha1* and *SPBC1711.09c* loci.
cdc2-asM17::bsdMX	A kind gift from Viestrus Simanis.	Evolved Cdc2 analogue sensitive allele. (Aoi et al., 2014)
cig1Δ::bsdMX	SmaI linearized **pAV0640** was used to obtain blasticidin-S resistant transformants	*cig1* ORF is replaced with pFA6a plasmid carrying *bsdMX* selection cassette.
cig2Δ::natMX	AfeI linearized pAV0646 was used to obtain nourseothricin resistant transformants	*cig2* ORF is replaced with pFA6a plasmid carrying *natMX* selection cassette.
crs1Δ::kanMX	SmaI linearized **pAV0642** was used to obtain G418 resistant clones	*crs1* ORF is replaced with pFA6a plasmid carrying *kanMX* selection cassette.
fus1-mCherry::kanMX	SmaI linearized **pAV0904** was used to obtain G418 resistant clones	Native *fus1* is fused with C-terminal mCherry and linked with *kanMX* selection cassette.
fus1-mCherry::kanMX::p^{tdh1}*:Pof1Nterminus-GBP:term*^{ScCYC1}	SmaI linearized **pAV0906** was used to obtain G418 resistant clones	Native *fus1* is fused with C-terminal mCherry and linked with the *kanMX* selection cassette and the construct for expresssion of DegGreen. DegGreen is comprised of the N-terminus of E3 ligase Pof1 (a.a. 1-261) fused to the GFP-binding protein (GBP) and expresssed under the regulation of the strong *p*^{td} promoter (1000bp upstream the START codon) and the budding yeast ADH1 transcriptional terminator.
fus1-meGFP::kanMX	SmaI linearized **pAV0903** was used to obtain G418 resistant clones	Native *fus1* is fussed with C-terminal meGFP and linked with the *kanMX* selection cassette.
fus1-meGFP::kanMX::p^{tdh1}*:Pof1Nterminus-ChBP:term*^{ScCYC1}	SmaI linearized **pAV0905** was used to obtain G418 resistant clones	Native *fus1* is fussed with C-terminal meGFP and linked with the *kanMX* selection cassette and the construct for expresssion of DegRed. DegRed is comprised of the N-terminus of E3 ligase Pof1 (a.a. 1-261) fused to the mCherry-binding (ChBP) protein and expresssed under the regulation of the strong *p*^{tdh1} promoter (1000bp upstream the START codon) and the budding yeast ADH1 transcriptional terminator.
fus1Δ::natMX	The natMX cassette from **pFA6a-natMX** (Hentges et al., 2005) was PCR amplified with primers **osm933**(ttttattaattataatttcattataatttgtttaagtcatttaattgtcattaaaagtcattaacatttcaaacatcaGAATTCGAGCTCGTTTAAAC) and **osm1670**(ttacgagcaaaaaaccttgtttctgtaattataggacatattattgatggtttcacctttttttagctattgcttgttaCGGATCCCCGGGTTAATTAA) that carry homology to the sequences flanking the fus1 ORF. The PCR product was used to obtain nourseothricin resistant clones.	*fus1* ORF is replaced by the *natMX* selection cassette
h90	*mat1* locus variant	Switching *mat1* mating type locus (Beach and Klar, 1984)
h-	*mat1* locus variant	M-cell locked *mat1* locus (Beach and Klar, 1984)
h+	*mat1* locus variant	P-cell locked *mat1* locus (Beach and Klar, 1984)
h?	heterothalic *mat1* locus identity unknown	heterothalic *mat1* locus identity unknown
h-::Mat1[Mi-sfGFP]H1$^{\Delta17}$*::kanMX*	PciI fragment of **pAV0551** was used to obtain G418 resistant transformants.	M-cell specific *mat1* locus carries the *H1Δ17* mutation in the H1-homolgy box, which prevents mating type switching. The *mi* encoded at the *mat1* locus is fused with the C-terminal sfGFP and inked with the *kanMX* resistance cassette
h-::Mat1[WT]H1$^{\Delta17}$*::natMX*	EcoRI fragment of **pAV0303** was used to obtain nourseothricin resistant transformants.	M-cell specific *mat1* locus carries the *H1Δ17* mutation in the H1-homolgy box, which prevents mating type switching. The locus is linked with the *natMX* resistance cassette
h+::Mat1[PiΔ]H1$^{\Delta17}$*::natMX*	EcoRI fragment of **pAV0353** was used to obtain nourseothricin resistant transformants.	P-cell specif *mat1* locus lacks the *pi* ORF and carries the *H1Δ17* mutation in the H1-homolgy box, which prevents matings type switching. The locus is linked with the *natMX* resistance cassette
h+::Mat1[WT]H1$^{\Delta17}$*::bleMX*	EcoRI fragment of **pAV0604** was used to obtain zeocin resistant transformants.	P-cell *mat1* locus carries the *H1Δ17* mutation in the H1-homolgy box, which prevents mating type switching. The locus is linked with the *bleMX* resistanc cassette
h+::Mat1[WT]H1$^{\Delta17}$*::natMX*	EcoRI fragment of **pAV0301** was used to obtain nourseothricin resistant transformants.	The P-cell specific *mat1* locus carries the *H1Δ17* mutation in the H1-homolgy box, which prevents mating type switching. The locus is linked with the *natMX* resistance cassette
his5+::p^{pcn1}*:eGFP-linker-pcn1:3'UTR*^{pcn1}*:terminator*^{NMT}*::bleMX*	StuI linearized **pAV0885** was used to obtain zeocin resistant transformants.	The eGFP is fused to the N-terminus of the S-phase marker Pcn1 and regulated by the *pcn1* promoter and the 3'UTR followed by the *nmt1* terminator. The construct is integrated at the *his5* locus and linked with the *bleMX* selection marker.
leu1-32::p^{SV40}*:GFP-atb2::leu1+*	Obtained from FC1234 strain (Minc *et al.*, 2009).	Construct for SV40 promoter driven expression of GFP-α-tubulin is integrated at the *leu1* locus.
leu1+::kanMX::p^{tdh1}*:sfGFP::apc10+*	StuI linearized **pAV0708** was used to obtain G418 resistant transformants.	sfGFP is expressed from the strong *p*^{tdh1} promoter, the construct linked to *kanMX* selection cassette and integrated in the intergenic region between *aha1* and *SPBC1711.09c* loci.

lys3+::p^{map3} *:mCherry::natMX*	SpeI linearized pAV0543 was used to obtain nourseothricin resistant transformants.	mCherry is expressed from the P-gamete specific p^{map3} promoter (2063bp upstream START codon), the construct integrated at the *lys3* locus and linked with the *natMX* selection cassette.
mei2Δ::hphMX	AfeI linearized **pAV0550** was used to obtain hygromycin resistant transformants.	*mei2* ORF is replaced with pFA6a plasmid carrying *hphMX* selection cassette.
mei2Δ::kanMX	Obtained from Bioneer library S20A08.	mei2 ORF replaced with *kanMX* selection cassette.
mei2^{F644A} *::hphMX*	AfeI linearized **pAV0443** was used to obtain hygromycin resistant transformants	The native *mei2* locus carries the F644A mutation linked with the *hphMX* selection cassette.
mei3Δ::bsdMX	The *bsdMX* cassette from **pAV0781** (pFA6a-bsdMX) was PCR amplified with primers **osm745**(GACATGGAGGCCCAGAATAC) and **osm746**(TGGATGGCGGCGTTAGTATC). The resulting PCR product, which carries homology to the *kanMX* flanking regions, was transformed into *mei3Δ::kanMX* strain to obtain G418-sensitive, blasticidin-S resistant transformants.	mei3 ORF replaced by the *bsdMX* selection cassette.
mei3Δ::hphMX	AfeI linearized **pAV0639** was used to obtain hygromycin resistant transformants.	*mei3* ORF was replaced with pFA6a plasmid carrying *hphMX* selection cassette.
mei3Δ::kanMX	Obtained from Bioneer library S07B02.	*mei3* ORF is replaced with G418 resistance cassette.
mei3Δ::mCherry::hphMX	SphI/PacI fragment of **pAV0398** was used to obtain hygromycin resistant transformants.	*mei3* ORF is replaced with mCherry linked to the *hphMX* resistance cassette.
mei3Δ::mei2^{ORF} *::hphMX*	AfeI linearized **pAV0603** was used to obtain hygromycin resistant transformants.	*mei3* ORF is replaced by the ORF of *mei2* and the *hphMX* selection cassette.
mei3Δ:puc1::hphMX	AfeI linearized **pAV0878** was used to obtain hygromycin resistant transformants.	*mei3* ORF is replaced by the ORF of *puc1* and linked with the *hphMX* selection cassette.
mei4Δ::ura4+	Obtained from National BioResource Project Yeast Strain Repository, Strain ID: FY7361	*mei4* ORF is replaced with ura4+ gene.
myo52-tdTomato::natMX	Obtained from ySM740 (Martin *et al., 2007)* .	Native *myo52* is fused with C-terminal tdTomato and linked with *natMX* resistance cassette.
pat1Δ::hphMX	AfeI linearized **pAV0528** was used to obtain hygromycin resistant transformants.	*pat1* ORF is replaced with the plasmid carrying the *hphMX* resistance cassette.
pat1Δ::natMX	AfeI linearized **pAV0556** was used to obtain nourseothricin resistant transformants.	*pat1* ORF is replaced with the plasmid carrying the *natMX* resistance cassette.
pcp1-mCherry::kanMX	AfeI/SnaBI fragment of **pAV0436** was used to obtain G418 resistant transformants.	Native *pcp1 is fused* with C-terminal mCherry and *kanMX* resistance cassete.
p^{mam2} *:GFP::ura4+::mam2+*	PmeI linearized **pSM1667** was used to obtain uracil auxotroph transformants	The GFP is placed under the regulation of the M-cell specific *p* ^{mam2} pror (438bp upstrem the START codon) and integrated at the *mam2* genomic locus.
puc1Δ::bleMX	AfeI linearized **pAV0641** was used to obtain zeocin resistant transformants	*puc1* ORF is replaced with pFA6a plasmid carrying *bleMX* selection cassette.
rem1Δ::kanMX	Obtained from Bioneer library S17H06.	*rem1* ORF is replaced by the *kanMX* selection cassette.
rlc1-sfGFP::natMX	The sfGFP with natMX cassette was PCR amplified from pSM1686 with primers osm2880(ttacgctcgtacaccagtcatggtgtattctattacgagaaattcgttgatgctattgctgggtcaaaagatagcaatCGGATCCCCGGGTTAATTAA) and osm2881(cgtctaagggaaatggctcaggttaaaaagataaagtattagagggaagaatgtgaaacatatctggctgctcttaacGAATTCGAGCTCGTTTAAAC) that carry homology to the sequences flanking the rlc1 STOP codon. The PCR product was ussed to obtain nourseothricin resistant transformants.	Native *rlc1* is fused with C-terminal sfGFP and linked with *natMX* resistanc cassete.
scd2-eGFP::patMX	AfeI linearized **pAV0892** was used to obtain glufosinate-ammonium resistant transformants.	Native *scd2* is fussed with C-terminal eGFP and linked with the *patMX* selection cassette.
scd2-GFP::hphMX	Obtained from ySM2477 (Bendezú *et al.* , 2015).	Native *scd2* is fused with the C-terminal GFP linked to *hphMX* resistance.
sme2Δ::ura4+	Obtained from National BioResource Project Yeast Strain Repository, Strain ID: FY7237	*sme2* ORF is replaced with *ura4+* gene.
uch2-mCherry::natMX	AfeI linearized **pAV0358** was used to obtain nourseothricin resistant transformants.	Native *uch2* is fused to C-terminal mCherry and linked with *natMX* resistance cassete.
ura4+::p^{mei3} *:mei2*^{ORF} *::hphMX*	AfeI linearized **pAV0627** was used to obtain hygromycin resistant transformants.	Mei2 is expressed from the zygote-specific *p*^{mei3} promoter (1054bp upstream START codon), linked with hphMX selection cassete and integarted at the *ura4* locus.
ura4+::p^{pcn1} *:eGFP-linker-pcn1:3'UTR*^{pcn1} *:terminator*^{nmt} *::bsdMX*	AfeI linearized **pAV0918** was used to obtain blasticidin-S resistant transformants.	The eGFP is fused to the N-terminus of the S-phase marker Pcn1 and regulated by the *pcn1* promoter and the 3'UTR followed by the *nmt1* terminator. The construct is integrated at the *ura4* locus and linked with the *bsdMX* selection cassette.
ura4+::p^{pcn1} *:eGFP-linker-pcn1:3'UTR*^{pcn1} *:terminator*^{NMT} *::natMX*	AfeI linearized **pAV0620** was used to obtain blasticidin-S resistant transformants.	The eGFP is fused to the N-terminus of the S-phase marker Pcn1 and regulated by the *pcn1* promoter and the 3'UTR followed by the *nmt1* terminator. The construct is integrated at the *ura4* locus and linked with the *natMX* selection cassette.
ura4+::p^{tdh1} *:Pof1Nterminus-ChBP:terminator*^{ScCYC1}	AfeI linearized **pAV0734** was transformed into *ura4-D18* mutant followed by selection for uracil prototrophs	The DegRed construct. The N-terminus of E3 ligase Pof1 (a.a. 1-261) is fused to mCherry-binding protein (ChBP) and expresssed under the regulation of the strong *p*^{tdh1} promoter (1000bp upstream the START codon) and the budding yeast ADH1 transcriptional terminator. The construct is integrated at the *ura4* locus.
ura4+::p^{tdh1} *:Pof1Nterminus-GBP:terminator*^{ScCYC1}	AfeI linearized **pAV0728** was transformed into *ura4-D18* mutant followed by selection for uracil prototrophs	The DegGreen construct. The N-terminus of E3 ligase Pof1 (a.a. 1-261) is fused to the GFP-binding protein (GBP) and expresssed under the regulation of the strong *p*^{tdh1} promoter (1000bp upstream the START codon) and the budding yeast ADH1 transcriptional terminator. The construct is integrated at the *ura4* locus.
ura4+::p^{tdh1*} *:mCherry:terminator*^{tdh1}	AfeI linearized **pAV0473** was transformed into *ura4-D18* mutant followed by selection for uracil prototrophs	mCherry is under the regulation of the strong *p*^{tdh1*} promoter (896bp upstream START codon) and integrated at the *ura4* locus.
ura4+::p^{tdh1*} *:mei3:terminator*^{tdh1}	**pAV0423** was partially digested with AfeI and transformed into *ura4-D18* mutant followed by selection for uracil prototrophs.	Mei3 is under the regulation of the strong, constitutive *p*^{tdh1} promoter (896bp upstream START codon) and integrated at the *ura4* locus.
ura4+::p^{tdh1*} *:sfGFP:terminator*^{tdh1}	AfeI linearized **pAV0569** was transformed into *ura4-D18* mutant followed by selection for uracil prototrophs	sfGFP is is under the regulation of the strong *p*^{tdh1*} promoter (896bp upstream START codon) and integrated at the *ura4* locus.
